# Selection of suitable reference genes for gene expression studies in HMC3 cell line by quantitative real-time RT-PCR

**DOI:** 10.1038/s41598-024-52415-7

**Published:** 2024-01-29

**Authors:** Martina Fazzina, Matteo Bergonzoni, Francesca Massenzio, Barbara Monti, Flavia Frabetti, Raffaella Casadei

**Affiliations:** 1 Department for Life Quality Studies - QUVI, University of Bologna, Rimini, Italy; 2 Department of Pharmacy and Biotechnology - FABIT, University of Bologna, Bologna, Italy; 3 Department of Medical and Surgical Sciences - DIMEC, University of Bologna, Bologna, Italy

**Keywords:** Microglia, Transcriptomics

## Abstract

Microglia represent the primary immune defense system within the central nervous system and play a role in the inflammatory processes occurring in numerous disorders, such as Parkinson’s disease (PD). PD onset and progression are associated with factors considered possible causes of neuroinflammation, i.e. genetic mutations. In vitro models of microglial cells were established to identify specific molecular targets in PD through the analysis of gene expression data. Recently, the Human Microglial Clone 3 cell line (HMC3) has been characterized and a new human microglia model has emerged. Here we perform RT-qPCR analyses to evaluate the expression of ten reference genes in HMC3, untreated or stimulated to a pro-inflammatory status. The comparative ∆C_T_ method, BestKeeper, Normfinder, geNorm and RefFinder algorithms were used to assess the stability of the candidate genes. The results showed that the most suitable internal controls are *HPRT1*, *RPS18 and B2M* genes. In addition, the most stable and unstable reference genes were used to normalize the expression of a gene of interest in HMC3, resulting in a difference in the statistical significance in cells treated with Rotenone. This is the first reference gene validation study in HMC3 cell line in pro-inflammatory status and can contribute to more reliable gene expression analysis in the field of neurodegenerative and neuroinflammatory research.

## Introduction

A growing body of evidence suggests that microglia, the primary cells involved in immune function within the Central Nervous System (CNS), play a role in the onset of numerous CNS disorders, including Parkinson’s disease (PD)^1,2^.

Two of the main risk factors for PD, aging and chronic stress, are linked to increased levels of pro-inflammatory mediators both in the peripheral and central nervous system^3,4^. Microglia have been suggested to mediate the inflammatory response at CNS level in Parkinson’s disease conditions. Nevertheless, the implication of microglia in the onset and progression of PD is still unclear, and it is yet to be undetermined whether their alterations are a cause or consequence of the loss of dopaminergic (DA) neurons.

To date, several factors have been identified as possible causes, including environmental factors^5,6^ and mutations in susceptibility loci associated with both the Mendelian and the sporadic form of the disease^7^. Furthermore, epigenetic mechanisms have been observed to regulate neuronal pathways in both types of PD^8^ and recent studies emphasizes the critical role of non-coding RNAs (ncRNAs) in the pathogenesis of the disorder^9,10^.

Latest investigations have revealed that PD-associated genes are expressed not only in neurons, but also in glial cells, and play important functions in microglia and astrocytes^11^.

To identify specific molecular targets, a huge amount of gene expression data was obtained by quantitative real-time RT–PCR (RT-qPCR) analyses of in vitro PD models, like neuroblastoma SH-SY5Y^12,13^ and human monocytes THP-1 differentiated with PMA or murine microglia BV-2^14–16^.

Recently, a new microglia cell line has emerged, the Human Microglial Clone 3 cell line (HMC3) commercially available and authenticated by the American Type Culture Collection (ATCC^®^) since 2018^17^.

It is important to note that, over the past four years, the use of the HMC3 cell line has considerably increased, with over 100 papers retrieved on PubMed using “(HMC3) OR (HMC-3)” as search terms, including several related to PD and/or other neurodegenerative diseases^18–22^.

To obtain microglia activation simulating the classical activation state, the HMC3 cell line can be subjected to various pro-inflammatory stimuli, such as Interferon-γ (INF-γ) boosted with glucose^19^, 6-hydroxidopamine (6-OHDA) or 1-methyl-4-phenyl-1,2,3,6-tetrahydropyridine (MPTP) or Rotenone^23–25^.

RT-qPCR performed on treated or untreated HMC3 cells could be extremely valuable in assessing the differential expression of well-known established as well as new markers of inflammation and oxidative stress associated to PD onset and progression.

RT-qPCR is widely used in biological and medical research as it is a relatively simple procedure with the advantages of quantitative accuracy, high sensitivity and rapid reaction.

However, many variables, including RNA extraction yield, reverse transcription and amplification efficiency, could influence RT-qPCR accuracy^26^. Therefore, the gene expression levels require normalization using reference genes (RGs) to obtain reliable data. The identification of appropriate RGs is a crucial stage involved in this approach, to remove most of the technical variation in cDNA concentrations between samples.

Studies performed in 2002 by Vandesompele et al. and many others since then, highlight the risk of blindly relying on the assumption of stable expression of reference genes^27–29^. Instead of using the most commonly RGs, typically cellular maintenance genes like glyceraldehyde-3-phosphate dehydrogenase (*GAPDH*), actin beta (*ACTB*) and ribosomal protein S18 (*RPS18*), it is important to define the most consistent expressed genes compared to a selected gene of interest (GOI) in different tissues, treatments or cell types.

To date, no systematic validation of RGs has been performed in HMC3 microglial cell line, except for a recent study^30^, where sixteen RGs have been tested only in untreated HMC3. Other gene expression analysis in HMC3 showed normalized results with only one or two common RGs, without specifying any previous internal statistical validation^18,21,31^.

Here, we report the validation of ten reference genes to identify the most suitable internal control for normalization of RT-qPCR data in HMC3 under different pro-inflammatory conditions.

The candidate genes were: *ACTB*, *GAPDH*, *TBP* (TATA-box binding protein TBP), *HPRT1* (hypoxanthine phosphoribosyltransferase 1), *RPL13* (ribosomal protein L13), *HMBS* (hydroxymethylbilane synthase), *TMEM199* (transmembrane protein 199), *YWHAZ* (tyrosine 3-monooxygenase/tryptophan 5-monooxygenase activation protein zeta), *B2M* (β-2-microglobulin) and *RPS18* (ribosomal protein S18).

We analyzed the expression data using different independent statistical algorithms: the ΔC_T_ method^32^, GeNorm^27^, NormFinder^33^, BestKeeper^34^ and RefFinder^35^. In addition, the expression profile of a GOI was tested to verify our results. We selected the interleukin-1 beta (*IL-1β*) gene, a typical pro-inflammatory cytokine synthesized and released by activated microglia known to be upregulated under oxidative stress conditions induced by rotenone treatment^36,37^.

This study offers a valuable benchmark for gene expression research and can be used as a model for future investigations using the HMC3 microglial cell line.

## Results

### Expression levels of the candidate RGs

The ten selected reference genes, including corresponding GenBank accession numbers, primer sequences, and specific function are listed in Table 1.

**Table 1:** List of selected candidate reference genes and the gene of interest (GOI) analysed in the HMC3 cell line.

Gene symbol	GenBank accession number	Gene function (GO terms)	Primer pair (5´ → 3´)
*ACTB*	NM_001101.5	Structural constituent of cytoskeleton	AGACCTGTACGCCAACACAGT
			AGTACTTGCGCTCAGGAGGA
*B2M*	NM_004048.4	Peptide antigen assembly with MHC class II protein complex	GCGGGCATTCCTGAAGCTGACAGCA
			CTTTCCATTCTCTGCTGGATGACG
*GAPDH*	NM_002046.7	Glyceraldehyde-3-phosphate dehydrogenase (NAD^+^) (phosphorylating) activity	CAACGACCACTTTGTCAA
			CTGTGAGGAGGGGAGATTCA
*IL-1β*	NM_000576.3	Cytokine with pro-inflammatory activity	TGATGGCTTATTACAGTGGCAATG
			GTAGTGGTGGTCGGAGATTCG
*HMBS*	NM_000190.4	Hydroxymethylbilane synthase activity	CGGCTGCAACGGCGGAAGA
			GTCCCCTGTGGTGGACATAGC
*HPRT1*	NM_000194.3	Hypoxanthine phosphoribosyltransferase activity	CAGCCCTGGCGTCGTGATTAGT
			ATCTCGAGCAAGACGTTCAG
*RPL13*	NM_000977	Structural constituent of ribosome	TGAAGGAGTACCGCTCCAAAC
			GGAGACTAGCGAAGGCTTTGA
*RPS18*	NM_022551	Structural constituent of ribosome	CAGAAGGATGTAAAGGATGG
			TATTTCTTCTTGGACACACC
*TBP*	NM_003194.5	RNA polymerase II general transcription initiation factor activity	GTGATCTTTGCAGTGACCCAG
			CTGGAACTCGTCTCACTATTCA
*TMEM199*	NM_152464.3	Lysosomal protein catabolic process	CCTCCACGGAACCCAGAACTA
			TGAAGATGGTGATGACCAGAGC
*YWHAZ*	NM_001135699	Phosphoserine residue binding	TCCCGTTTCCGAGCCATAAA
			TGACCTACGGGCTCCTACAA

The specificity of the primers for all the candidate genes was first evaluated by endpoint PCR (data not shown) and confirmed by melt curves analyses (see Supplementary Fig. S1).

We used RT-qPCR to analyze the transcriptional expression levels of ten RGs in samples taken from the HMC3 microglia cell line exposed to three distinct oxidative and inflammatory stress molecules compared to untreated controls, as detailed in the "Methods" section.

Analysis of the data obtained as quantification cycle (C_q_), showed a variable expression for each RG in the different treatments. All the raw C_q_ data are listed in Supplementary Table S2.

**Figure 1:**
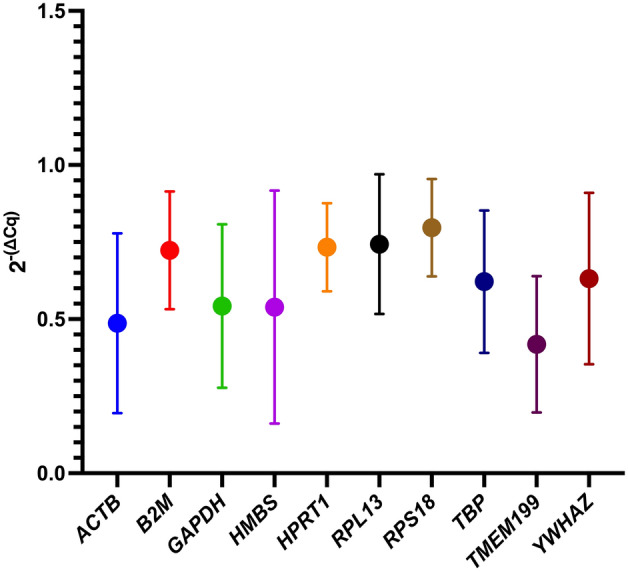
Frequency distribution of expression levels (2^−(∆Cq)^) calculated in ten candidate reference genes. The expression levels of ten candidate reference genes were evaluated in HMC3 control samples compared to treated samples (6-OHDA 1 µM, IFN-γ 1 µg/mL + glucose 5 g/L, Rotenone 0.1 µM). The data refer to three independent biological replicates. For each sample the arithmetic mean of the technical triplicates was considered.

In Fig. 1 we reported the fold-change values calculated for ten RGs as Q = 2^−(∆Cq)^. ∆C_q_ was calculated as [C_q sample_ − C_q min_] for each reference gene in all experimental conditions (see "Methods" section and Supplementary Tables S3 and S6a). A statistical frequency distribution test was conducted using GraphPad Prism 9 software, to visually represent the gene expression distribution of the putative reference genes analysed.

### Stability of the candidate RGs

A stability analysis of ten candidate reference genes in the chosen model, the human microglia line HMC3, was conducted using the following analysis tools: ∆C_T_ method, BestKeeper, Normfinder, geNorm and RefFinder. The obtained results are all shown in Table 2, each considering the stability parameter of each platform.

**Table 2:** Stability values determined by ∆C_T_ method (Mean SD values), BestKeeper (SD values), Normfinder (stability values), geNorm (M values) and RefFinder (Geomean), for ten candidate reference genes in the HMC3 microglia cell line across all the experimental samples.

Gene	∆C_T_ (SD)	BestKeeper (SD)	Normfinder (stability value)	geNorm (M value)	RefFinder (Geomean)
*ACTB*	1.29	0.96	0.378	0.6639	9.74
*B2M*	0.93	0.34	0.140	0.7068	3.87
*GAPDH*	1.51	0.98	0.315	1.2049	8.74
*HMBS*	1.21	1.09	0.532	0.8739	8.46
*HPRT1*	0.81	0.24	0.069	0.5613	1.68
*RPL13*	0.99	0.44	0.163	0.6326	4.36
*RPS18*	0.94	0.26	0.230	0.4765	1.86
*TBP*	0.98	0.49	0.229	0.5032	2.00
*TMEM199*	1.03	0.52	0.298	0.5528	5.96
*YWHAZ*	0.96	0.65	0.199	0.5262	6.74

#### Comparative ∆C_T_ method

We used the comparative ∆C_T_ method to assess the relative expression of pairs of genes within each sample to determine which genes are suitable for expression analysis. After comparing ∆C_T_ and Standard Deviation (SD) values, obtained from raw C_q_ data, it will be possible to determine which ones exhibit less fluctuation and the more stable expression. In particular, analyzing across several samples and conditions, if the ∆C_T_ value remains constant, it indicates that both genes are expressed similarly in those samples. However, if the ∆C_T_ changes, one or both genes demonstrate variable expression^32^.

In Table 2 we reported the average SD values for each RG across all the HMC3 samples. The genes with the lowest SD values are the least variable, while those with the highest values are considered the most unstable. For example, *HPRT1* and *B2M* showed a smaller standard deviation compared to *ACTB* and *GAPDH*, which display the highest variation. Supplementary Table S4 reports the overall ∆C_T_ data.

#### BestKeeper

BestKeeper software performs a calculation based on the averages of the calculated raw data (C_q_), returning the following statistical parameters: the standard deviation (SD), the variance coefficient, and the BestKeeper Index for each of the ten reference genes^34^.

Table 2 lists the SD values, the parameter chosen to determine the ideal combination of reference genes to be employed for RT-qPCR analyses of certain target genes. Genes with SD values greater than 1 are deemed unstable and cannot be used as reference genes.

The overall results obtained with Bestkeeper are shown in Supplementary Table S5: *HMBS* has SD value > 1, therefore it is considered the least stable gene, to be excluded in subsequent normalization analyses. The most stable genes resulted *HPRT1* and *RPS18*.

#### Normfinder

The Normfinder software's algorithm can determine the intra-group and inter-group variation, or more specifically, the variance between biological samples from the same treatment group and from other treatment groups^33^. Combining these two types of analyses yields a result known as the "stability value", which can be used to choose the optimum housekeeping pair and reference genes for upcoming expression analyses.

We performed two types of analyses with Normfinder: the first by setting subgroups of experimental conditions and biological replicates, the second without specifying the subgroups and therefore the different experimental conditions, to highlight any software calculation differences. Table 2 shows the stability value ​​returned by Normfinder with the first type of setting. The data relating to the second type of analysis are shown in the Supplementary Table S6b, together with the Q values for each RGs, calculated via the formula Q = 2^−(∆Cq)^, as described in the "Methods" section (Supplementary Table S6a). The most stable genes have the lower stability value. In both analyses the best two RGs resulted *HPRT1* and *B2M.*

#### GeNorm

GeNorm algorithm calculates the average pairwise expression ratio to evaluate expression stability, and genes with lower stability measure (M) values were more stable.

According to geNorm analysis, the M-values of all tested RGs, except *GAPDH*, were below 1, indicating that they were relatively stable, with the lowest M-value associated to the highest stability.

CFX Maestro Software employs the geNorm algorithm, which is detailed in Vandesompele’s study^27^. For homogenous samples, an M-value less than 0.5 is considered “stable”. Our results highlight only one gene with such a M-value, *RPS18* (Table 2).

#### RefFinder

We assessed the stability of RGs with the free web tool RefFinder, which integrates all the other algorithms^35^. The RefFinder software comprehensively determines the overall ranking of the tested RGs based on the geometric mean of the weights of every gene calculated by each program. Raw C_q_ values (untransformed data) were used for RefFinder data import. Stability values indicated as Geomean are listed in Table 2.

The stability ranking of the ten RGs defined by RefFinder software is shown in Table 3.

**Table 3:** Expression stability ranking of ten RGs in HMC3 cell line according to RefFinder analysis.

	1	2	3	4	5	6	7	8	9	10
∆C_T_ method	*TBP*	*HPRT1*	*RPS18*	*RPL13*	*B2M*	*TMEM199*	*YWHAZ*	*HMBS*	*GAPDH*	*ACTB*
BestKeeper	*RPS18*	*HPRT1*	*B2M*	*TBP*	*TMEM199*	*RPL13*	*YWHAZ*	*GAPDH*	*ACTB*	*HMBS*
Normfinder	*TBP*	*HPRT1*	*RPL13*	*RPS18*	*B2M*	*YWHAZ*	*TMEM199*	*HMBS*	*GAPDH*	*ACTB*
geNorm	*HPRT1*	*RPS18*	*B2M*	*TBP*	*RPL13*	*YWHAZ*	*TMEM199*	*HMBS*	*GAPDH*	*ACTB*
Recommended comprehensive ranking	*HPRT1*	*RPS18*	*TBP*	*B2M*	*RPL13*	*TMEM199*	*YWHAZ*	*HMBS*	*GAPDH*	*ACTB*

### Comprehensive ranking and validation of the selected RGs

To obtain an overall ranking that takes into account each analysis, we calculated the geometric mean of each RG position obtained in every individual program (Table 4).

**Table 4:** Expression stability ranking of ten RGs in HMC3 cell line:independent analyses and comprehensive ranking using geometric mean of ranks.

Ranking	∆C_T_ (SD)	BestKeeper (SD)	Normfinder (Stability value)	geNorm (M value)	RefFinder (Geomean)	Final ranking (Geomean)
1	*HPRT1*	*HPRT1*	*HPRT1*	*RPS18*	*HPRT1*	*HPRT1*
2	*B2M*	*RPS18*	*B2M*	*TBP*	*RPS18*	*RPS18*
3	*RSP18*	*B2M*	*RPL13*	*YWHAZ*	*TBP*	*B2M*
4	*YWHAZ*	*RPL13*	*YWHAZ*	*TMEM199*	*B2M*	*TBP*
5	*TBP*	*TBP*	*TBP*	*HPRT1*	*RPL13*	*RPL13*
6	*RPL13*	*TMEM199*	*RPS18*	*RPL13*	*TMEM199*	*YWHAZ*
7	*TMEM199*	*YWHAZ*	*TMEM199*	*ACTB*	*YWHAZ*	*TMEM199*
8	*HMBS*	*ACTB*	*GAPDH*	*B2M*	*HMBS*	*ACTB*
9	*ACTB*	*GAPDH*	*ACTB*	*HMBS*	*GAPDH*	*HMBS*
10	*GAPDH*	*HMBS*	*HMBS*	*GAPDH*	*ACTB*	*GAPDH*

**Figure 2:**
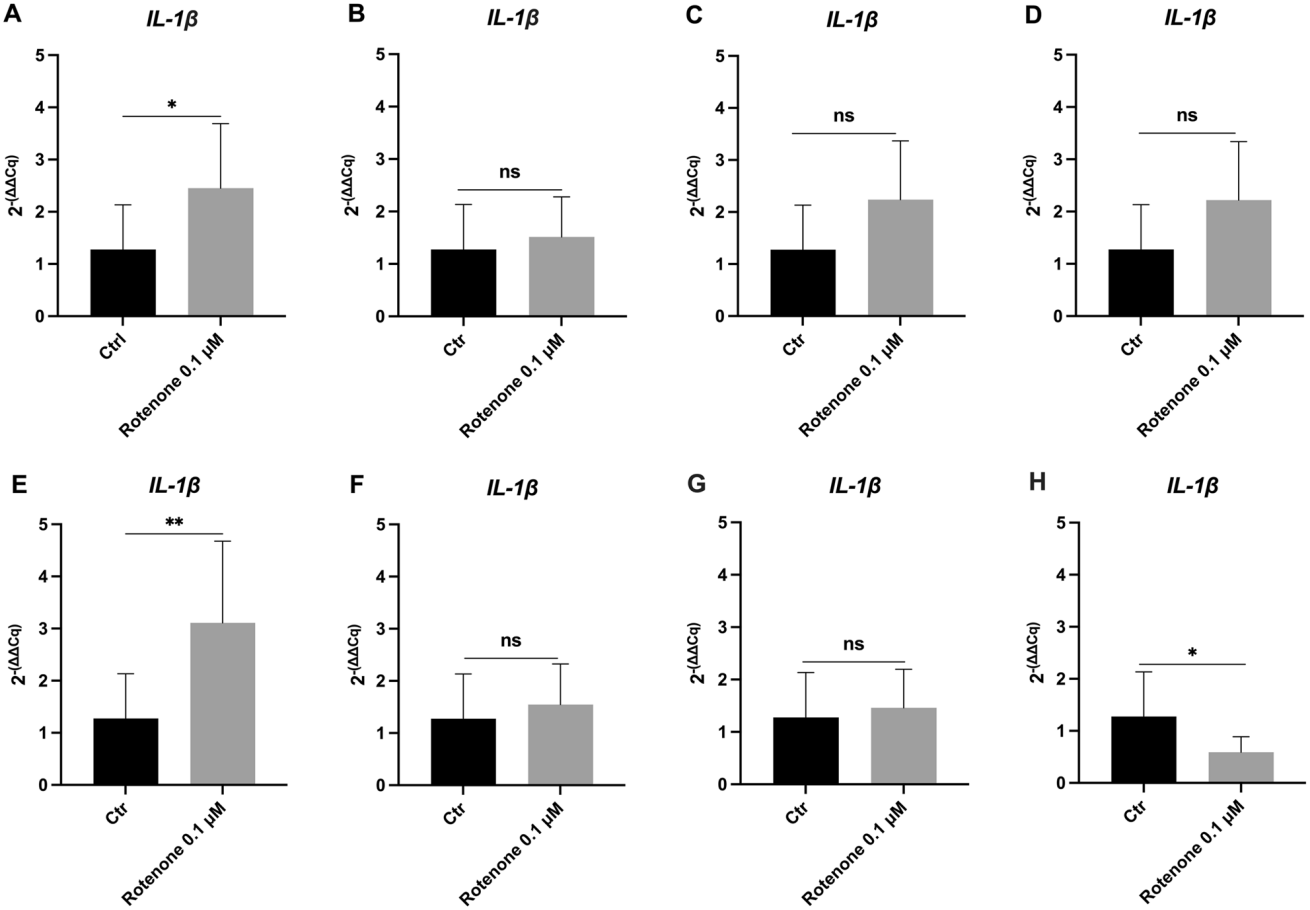
Gene expression analysis of *IL-1β* in HMC3 cell line exposed to Rotenone treatment. (**A**,**B**) normalization with the combination of the top three-ranked reference genes (*HPRT1*, *RPS18*, *B2M*) and the worse three reference genes (*GAPDH*, *HMBS, ACTB*) respectively, according to the overall analysis; (**C**–**H**) normalization with the three best and worst single RGs, *HPRT1* (**C**) and *GAPDH* (**D**)*, RPS18* (**E**) and *HMBS* (**F**)*, B2M* (**G**) and *ACTB* (**H**) respectively. Data are expressed as fold-change values (2^−(ΔΔCq)^) in reference to untreated control samples. Student’s t-test (unpaired, two-tailed), *p < 0.05; **p < 0.01; *ns* not significant vs. Ctr.

Finally, to experimentally validate our overall ranking results, we considered a common marker of activated microglia, the *IL-1β* gene, whose overexpression data after rotenone-induced oxidative stress are well known^36,37^. The expression level of *IL-1β* mRNA was evaluated in untreated and rotenone-stimulated HMC3 cells, and values were normalized with different assays using the combination of the most stable candidate reference genes (*HPRT1, RPS18, B2M*) versus the most unstable reference genes (*GAPDH, HMBS, ACTB*), as well as the best and worse 3 individual top ranked candidates (Fig. 2).

The results showed a statistically significant up-regulation of the GOI in HMC3 cells treated with Rotenone 0.1 µM for 24 h compared to untreated cells, normalized with the three top-ranked genes (Fig. 2A, p < 0.05). The same expression trend was not appreciable in the normalization with the combination of the three worst genes, where a very similar trend is observed between the two conditions analyzed (Fig. 2B). Normalization with the best and worst individual RG indicated a similar trend of *IL-1β* upregulation in rotenone-treated cells, although without statistical significance (Fig. 2C,D). In the normalization analysis using only *RPS18* or *HMBS* another significant set of data was identified, but with a considerably distinct pattern of expression in the treated sample (Fig. 2E,F, p < 0.01).

## Discussion

The role of glial cells in the central nervous system (CNS) has been the subject of numerous studies. Only a few years after its discovery, microglial cells were found to play a significant role in both physiological and pathological neuronal systems.

Microglia represent the immune cells of the brain and are considered the neural tissue’s defense system^38^. In the CNS, they constitute up to 12% of all cells and their quantity varies depending on the brain region considered^39^. Microglial cells are essential in both development and adult physiology: during development, they regulate synaptic transmissions and the growth of neuronal synapses contributing to the formation and refinement of neuronal circuits^40,41^; in the adult brain, microglia not only continuously monitor the physiopathological state of the synapses, but also could become activated and secrete many soluble factors, such as chemoattractant, cytokines, and neurotrophic factors that contribute to various aspects of immune responses and tissue repair in the CNS^42,43^. According to emerging genetic and functional evidence, when microglial cell activities are impaired, they are mainly involved in the pathogenesis of neurodegenerative diseases^44–46^. It is now established that even microglia can adopt a pathogenic and activated phenotype, leading to the progression of neuronal damage as well as the spread of inflammation and oxidative stress. However, whether microglia have beneficial or harmful functions in this context is controversial, although a context-specific role has been suggested^47^.

Although rodent models of microglia are widely used, limited overlap was observed in microglial genes regulated during aging and in response to human inflammatory conditions between rodents and humans, suggesting divergences between species^48,49^. The identification of these dissimilarities, along with the associated biological properties might explain the failure of human trials when microglia is targeted. However, the scarce availability of primary sources of human microglia limits the research in the field. Furthermore, human microglial cell lines can be considered a valuable in vitro model to gain more insight into the functions and roles of microglia in the CNS. The human microglial clone 3 cell line, HMC3, was established in 1995, through SV40-dependent immortalization of human embryonic microglial cells^50^.

It has been characterized by low to absent expression of CD14 and CD11b, similarly to human iPSC-derived microglia, positive for both macrophage markers IBA1 and Cx3cr1, and microglial ones TMEM119, P2RY12, and TREM2 corroborating the value of the cell model^51,52^.

It has been recently authenticated by the American Type Culture Collection (ATCC^®^) and is available for purchase under the recognized name HMC3 (ATCC®CRL-3304).

The use of this human cell line has only recently started to take off; in fact, by searching with "HMC3" or "HMC-3" on PubMed, 134 results are from 2019 to 2023, of which more than half published starting from 2022 until today.

We employed HMC3 cell line to perform gene expression studies following treatments that induce oxidative and inflammatory stress, observable in neurodegenerative contexts such as PD. However, we immediately noticed a high variance in both untreated and treated cells, not only in the expression of target genes, but also of housekeeping genes some of which are the most used for relative quantitative gene expression analyses.

RT-qPCR technique is the method of choice for gene expression studies due to its high sensitivity and precision and its wide range of quantification in biological samples. Given the high sensitivity of RT-qPCR, an internal control known as a reference gene is required to normalize the gene expression level of a gene of interest. A reference gene should ideally have stable expression levels that do not differ significantly between samples and experimental conditions^27,28^.

To date, only one stability study of selected RGs in the HMC3 cell line has been conducted in a different pathological context, namely glioblastoma^30^. The authors analyzed the stability of sixteen RGs that are mostly involved in metabolic pathways associated with pediatric glioma, using untreated HMC3 cells as a model system to find the most suitable genes for intended expression analysis in glioma samples^30^.

Since our goal is to investigate the role of activated microglia in a pro-inflammatory status, we conducted a stability analysis in untreated control cells and cells subjected to stressogenic treatments, such as 6-OHDA, Rotenone and Interferon-γ. We selected ten RGs, starting from some of the most common housekeeping genes in expression studies*,* related to basic cellular functions or components, like glycolysis (*GAPDH*), transcription initiation (*TBP*), cytoskeleton (*ACTB*) histocompatibility complex (*B2M*) and ribosome (*RPS18*). Then, considering the glial nature of HMC3 we focused on other genes constitutively expressed in CNS and/or immune system cells, like *HPRT1*^53^, *TMEM199*^54^, *RPL13*^55^, *YWHAZ*^56^ and *HMBS*^57^.

The stability of the reference genes was evaluated in different samples of treated and untreated HMC3 cells using five algorithms, namely comparative ∆C_T_ method, BestKeeper, Normfinder, geNorm and RefFinder. The tools have distinct methods of processing the expression data, and their respective interpretation of the results is based on specific statistical criteria, as indicated in the “Results” section.

RefFinder is a web tool that could provide an overall ranking based on the Geometric Mean (GM) values of the results from the other different statistical algorithms. It must be highlighted that different outputs could be obtained between the original software packages and the RefFinder tool, which is based on raw C_q_ values for input, while Normfinder and geNorm algorithm require the Q value (2^-(∆Cq)^) calculated from original C_q_ values. Comparing the data obtained from each software with those returned by the RefFinder platform could reveal slight differences. Despite the differences among the algorithms the outcome of the most stable and least stable reference genes is largely comparable for each sample sets^58^.

For this reason and trying to achieve even more significant data we conducted the analysis with every single method considered by RefFinder and we calculated an overall ranking based on the geometric mean of all five ranks (Table 4). In our honest opinion, this could provide a stable approach and a good baseline method rather than the use of one of these programs as inaccuracies and instabilities in outcomes could occur.

The geNorm result, for example, showed *HPRT1* as one of the middle-rank reference genes (Table 4), while all other algorithms indicate it as the best one (Table 4). Similarly, *B2M* is listed among the worst three candidates while, in every other analysis, it is indicated as very stable (ranking position from 2 to 4).

Nonetheless, according to our Normfinder analysis (see “Results” section), the gene *RPS18* is not one of the three most stable candidate genes, as for all the other statistical algorithms (Table 4). This result is also confirmed by the other type of Normfinder analysis which does not consider the subgroups of experimental conditions (Supplementary Table S6b).

It is significant to report that the most stable genes under these conditions are similar across all software programs, as are the less stable ones. This comforts us and makes it easier to select the RGs that will provide the best normalization in subsequent investigations. As a general guide, validation using a high number of candidate genes is always recommended.

According to our thorough investigation, the top three most stable reference genes for all treatments investigated were *HPRT1*, *RPS18* and *B2M*, whereas the three least stable genes were *GAPDH*, *HMBS* and *ACTB.*

It is noteworthy, although not surprising, that among the worst candidates we can find genes, such as *GAPDH* and *ACTB*, which are routinely employed as reference genes for normalizing expression data of target genes in all the different samples or experimental conditions, without further validation. Gene expression studies with human HMC3 microglia line are no exception; for example some of the most recent ones report analyses with only one reference gene, such as *GAPDH* ^18,20–22^, *ACTB*^31,59,60^ or a combination of the two^61^. We only identified one gene expression study with normalized data using *B2M* and *HPRT1*^62^.

In addition, our findings are supported by the only other work on HMC3, Hernández-Ochoa et al., who found that the *GAPDH* is not the most suitable housekeeping gene in untreated HMC3 cells^30^.

Finally, to further confirm the validity of the data obtained, we evaluated the expression of the *IL-1β* gene in cells treated with a pro-inflammatory stimulus (Rotenone 0.1 μM) and controls. All the expression values were normalized with different assays using the combination of the most stable candidate reference genes (*HPRT1, RPS18, B2M*) versus the most unstable reference genes (*GAPDH, HMBS, ACTB*), as well as the best and worst three individual top-ranked candidates (Fig. 2).

The results showed a statistically significant up-regulation of the *IL-1β* when normalized with the three top-ranked genes (Fig. 2A, p < 0.05). Conversely, normalization with the combination of the three worst genes resulted in a similar trend between treated and untreated conditions (Fig. 2B). Normalization with the best and worst individual reference genes showed a non-significant trend of *IL-1β* upregulation in treated cells (Fig. 2C,D). Notably, significant data were found in normalization analyses using only *RPS18* or *HMBS*, but with a distinct pattern of expression in the treated samples (Fig. 2E,F, p < 0.01).

Overall, this work confirms the importance of selecting appropriate reference genes for mRNA quantification by RT-qPCR, because the use of non-validated reference genes can lead to questionable results with potential data misinterpretation.

This can be considered the first comprehensive reference gene validation study in the HMC3 cell line in neurodegenerative and neuroinflammatory expression studies. It can provide a useful framework to perform future analyses as well as potentially rectifying erroneous outcomes in already published data.

## Method

### Cell culture and treatments

The human microglia clone 3 (HMC3) cell line was employed to evaluate the expression stability of ten candidate reference genes after inflammatory and oxidative stress treatments. The HMC3 line, distributed under the name of HMC3 Human Microglia Clone 3 (ATCC®CRL-3304), was purchased from *American Type Culture Collection (ATCC*^*®*^*)*. Microglial cells were cultured in Dulbecco's modified Eagle Medium (DMEM) High Glucose, supplemented with 10% heat inactivated FBS and 1% antibiotic penicillin/streptomycin (all reagents from Euroclone, Milano, Italy) at 37 °C, 5% CO_2_, in a humidified atmosphere. Cells were seeded in 100-mm Petri dishes until reaching the number of 1 × 10^6^ with a growth rate of 3–4 days. Then, cells were plated in 60-mm Petri dishes at a density of 350,000 cells per dish and grown under standard conditions for 24 h before treatments, to obtain an 80% confluency. Cells were exposed to Interferon-gamma (IFN-γ, Sigma-Aldrich St Louis, MO, United States) at 1 μg/mL boosted by glucose 5 g/L to induce an inflammatory response for 24 h, or Rotenone 0.1 μM or 6-hydroxydopamine (6-OHDA) 1 μM (Sigma-Aldrich St Louis, MO, United States) for 24 h in serum-free medium to evoke a general oxidative stress response. The experiments were repeated in three biological replicates for each condition; cell samples were at different passage numbers, ranging from 32 to 48.

### Candidate reference gene selections

We conducted an expression stability analysis identifying ten candidate reference genes for accurate RT-qPCR procedure in the HMC3 cell line, based on their biological function and considering the experimental conditions as described in detail above. Selected reference genes are involved in biological mechanisms that are essential for cell survival, including maintenance of cell structure and integrity, cell signaling and transduction processes, cell metabolism enzymes, ribosome structural proteins and transcription factors. Furthermore, we searched the literature to identify housekeeping genes mainly used for expression analysis in the central nervous system or in glia samples. The genes selected were the actin beta (*ACTB*), Beta-2-microglobulin (*B2M*), glyceraldehyde-3-phosphate dehydrogenase (*GAPDH*), hydroxymethylbilane synthase (*HMBS*), hypoxanthine–guanine phosphoribosyl transferase 1 (*HPRT1*), 60S ribosomal protein L13 (*RPL13*), 40S ribosomal protein S18 (*RPS18*), TATA-box binding protein (*TBP*), transmembrane protein 199 (*TMEM199*), and tyrosine 3-monooxygenase/tryptophan 5-monooxygenase activation protein zeta (*YWHAZ*). The selected RGs, with detailed function and respective primer pairs are shown in Table 1.

### Total RNA isolation and quality control

Each independent replication of the HMC3 cultures was subjected to total RNA extraction, which was carried out in two steps: first, 750 μL of TRIzol (Invitrogen, Carlsbad, CA, USA) was poured directly into the growing surface of the dish to homogenize the cells and isolate RNA into the aqueous layer. Next, the column-based PureLink RNA Mini Kit (Invitrogen, Carlsbad, CA, USA) was used to wash and elute high-quality RNA, following the manufacturer's protocol. The samples were additionally subjected to on-column DNase treatment with ~ 3U/μL of Dnase I enzyme (Invitrogen, Carlsbad, CA, USA). Total RNA quantification was performed using NanoDrop™ 1000 spectrophotometer (Thermo Scientific) and RNA quality was assessed at absorbance values of 280/260 nm and 230/260 nm. RNA integrity was then evaluated by 1% agarose gel electrophoresis in TAE buffer with SYBR™ Safe DNA Gel Stain (Invitrogen Carlsbad, CA, USA) and analyzed with the gel imaging system Gel Doc 2000 (Bio-Rad, Hercules, CA, USA).

### Design and validation of primer pairs

We examined ten candidate reference genes to test their stability in our experimental conditions, as described above. All primer pairs were designed according to MIQE's guidelines^63^. Primer design and their target specificity were preferentially based on the longest isoform, allowing the amplification of all transcripts encoded by each gene. In Table 1 we listed the principal information about primer pairs of ten housekeeping genes chosen for our expression stability studies in the HMC3 model. Here, we reported the oligonucleotide sequences used for target-specific amplification with the NM code of reference. Primer design was based on the mRNA sequences obtained from the GenBank database. Then we used the NCBI software Primer BLAST, both to verify target specificity and to exclude unintended pairing with other targets. The prediction of good amplification efficiency and the probability of primer dimer formation was evaluated with the aid of Amplify4 software (Bill Engels, 2015, University of Wisconsin). Then, the resulting amplicon length and the absence of non-specific bands were evaluated by endpoint PCR using an initial quantity of 50 ng of cDNA as a template derived from the HMC3 cell line and the BioMix™ Red (Meridian Bioscience—Bioline), according to the manufacturer's proceedings. The amplification protocol was the following: 94 °C for 2 min enabling the first template denaturation; then the amplification steps repeated for 25 cycles (94 °C for 30'', 60 °C for 30'') and a final step at 72 °C per 5 min. PCR products were separated onto 2% (*w/v*) agarose gel electrophoresis, stained with SYBR™ Safe DNA Gel Stain (Invitrogen Carlsbad, CA, USA) and finally observed using the gel imaging system Gel Doc 2000 (Bio-Rad, Hercules, CA, USA).

### Reverse transcription quantitative polymerase chain reaction (RT-qPCR)

RT-qPCR analysis was performed after total RNA isolation to test differences in the expression of candidate reference genes in HMC3 cell line subjected to inflammatory and oxidative stress conditions, as described above.

2 μg of HMC3-derived total RNA was reverse transcribed into cDNA with iScript™ cDNA Synthesis Kit (Bio-Rad). RT-qPCR was performed in Hard-Shell® 96-Well PCR Plates (Bio-Rad), each well containing 20 μl final volume with 10 μl SsoAdvanced Universal SYBR Green Supermix (Bio-Rad), 20 ng cDNA diluted 5 ng/μl and 0.3 μM of each primer, adjusting the final volume with H_2_O RNase free. Each sample, representing four experimental conditions, was loaded in three technical replicates to increase the statistical analysis accuracy.

The RT-qPCR was performed in the CFX ConnectTM real-time PCR Detection System (Bio-Rad Laboratories, Hercules, CA, USA) with an initial denaturation step of 30'' at 95 °C followed by 40 cycles of amplification (95 °C for 5'', 60 °C for 30''). Melting curve analysis was carried out in a range of 65 °C–95 °C with an increment of 0.5 °C every 5''. Quantification cycle (C_q_) data were obtained with CFX Manager software (Bio-Rad, version 2.1) adjusting the threshold using a semi-logarithmic scale. The raw data were therefore subjected to subsequent statistical analysis in order to identify the most stable reference genes in HMC3 model.

### Statistical analysis on gene expression stability

The C_q_ values obtained from RT-qPCR were calculated for each sample, treated and untreated control, as arithmetic mean of three technical replicates.

The C_q_ values of the ten selected reference gene were analyzed through RefFinder (https://www.ciidirsinaloa.com.mx/RefFinder-master/) software, a user-friendly web-based comprehensive tool developed for evaluating and screening reference genes from extensive experimental datasets^35^. It integrates four major computational programs (the ∆C_T_ method, Normfinder, BestKeeper and geNorm) calculating the geometric mean (geomean) for each reference gene to give the ranking index of stability. A lower index value indicates a higher stability of the reference gene.

We also performed an independent analysis with each statistical algorithm: the ∆C_T_ method, BestKeeper, Normfinder and geNorm, the latter using the CFX Manager software.

BestKeeper, is an Excel based free tool able to test up to ten candidate genes and combines them into an index of expression stability starting from raw C_q_ values. Then, the algorithm measures the coefficient of variance (CV) and the standard deviation (SD) between all the housekeeping data without considering the different experimental conditions^34^.

The ∆C_T_ method compares the relative expression of "pairs of genes" within each biological sample to evaluate the gene expression variation of the selected reference genes. By coupling the raw C_q_ values of a reference gene with one another, we determined the ∆C_q_ value and the mean SD for each pair of gene using Excel.

Normfinder combines the variance both within each group (intragroup variation) and between different groups (intergroup variation) of the sample set and then evaluates the expression stability of each candidate reference gene according to its stability value^34^. Input data must be in linear scale: starting from raw C_q_ values, we calculated the relative quantitative (Q value) using Excel, via the formula Q = 2^−(∆Cq)^, being ∆C_q_ = C_q sample_ − C_q min_. C_q sample_ is the C_q_ value of the reference gene in each considered experimental condition. C_q min_ indicates the lowest C_q_ value of this reference gene among each experimental condition.

Then, the expression stability measurement (M) value was calculated by the geNorm program for each candidate reference gene.

## Supplementary Information

Supplementary Information.

## Data Availability

The authors confirm that the data supporting the findings of this study are available within the article and its Supplementary Information.
