# Author Correction: Selection of suitable reference genes for gene expression studies in HMC3 cell line by quantitative real-time RT-PCR

**DOI:** 10.1038/s41598-025-87066-9

**Published:** 2025-01-28

**Authors:** Martina Fazzina, Matteo Bergonzoni, Francesca Massenzio, Barbara Monti, Flavia Frabetti, Raffaella Casadei

**Affiliations:** 1 Department for Life Quality Studies - QUVI, University of Bologna, Rimini, Italy; 2 Department of Pharmacy and Biotechnology - FABIT, University of Bologna, Bologna, Italy; 3 Department of Medical and Surgical Sciences - DIMEC, University of Bologna, Bologna, Italy

Correction to: *Scientific Reports* 10.1038/s41598-024-52415-7, published online 29 January 2024

The original version of this Article contained an error in the Method section, under the subheading ‘Statistical analysis on gene expression stability’, where the URL of the RefFinder software was incorrect.

As a result,

“The Cq values of the ten selected reference gene were analyzed through RefFinder (http://www.leonxie.com/referencegene.php) software, a user-friendly web-based comprehensive tool developed for evaluating and screening reference genes from extensive experimental datasets^35^.”

now reads:

“The Cq values of the ten selected reference gene were analyzed through RefFinder (https://www.ciidirsinaloa.com.mx/RefFinder-master/) software, a user-friendly web-based comprehensive tool developed for evaluating and screening reference genes from extensive experimental datasets^35^.”

The original Article has been corrected.

